# Laparoscopic splenopancreatectomy for an endocrine tumor with cystic changes: a case report

**DOI:** 10.1002/ccr3.844

**Published:** 2017-02-06

**Authors:** Hideki Izumi, Naoki Yazawa, Daisuke Furukawa, Yoshihito Masuoka, Misuzu Yamada, Taro Mashiko, Yohei Kawashima, Masami Ogawa, Yoshiaki Kawaguchi, Tetsuya Mine, Kenichi Hirabayashi, Toshio Nakagohri

**Affiliations:** ^1^ Department of Gastrointestinal SurgeryTokai University School of Medicine143 ShimokasuyaIseharaKanagawa259‐1193Japan; ^2^ Department of Internal MedicineTokai University School of Medicine143 ShimokasuyaIseharaKanagawa259‐1193Japan; ^3^ Department of PathologyTokai University School of Medicine143 ShimokasuyaIseharaKanagawa259‐1193Japan

**Keywords:** Cystic change, laparoscopic splenopancreatectomy, nonfunctioning endocrine tumor of pancreas

## Abstract

The biological behavior of a cystic pancreatic endocrine neoplasm is less aggressive than that of pancreatic neuroendocrine neoplasms, and its prognosis is better. Limited surgery should be considered for cystic pancreatic endocrine neoplasms that are not accompanied preoperatively by lymph node or distant metastasis.

## Introduction

Pancreatic neuroendocrine neoplasms (PENs) are rare neoplasms that comprise only 5% of all pancreatic malignancies 1. PENs usually appear radiologically as solid tumors but may rarely manifest as cystic lesions of the pancreas 2. Cystic pancreatic endocrine neoplasms (CPENs) have been reported to represent 11–17% of resected PENs 3, 4, 5. In this study, which described laparoscopic splenopancreatectomy for a CPEN, the biological behavior of the CPEN was found to be less aggressive than that of PENs.

## Case Presentation

In 2009, a 70‐year‐old man was diagnosed with a pancreatic neoplasm via abdominal ultrasonography during a complete physical examination. He then underwent regular follow‐up examinations with his local general practitioner. In October 2014, he was referred to Tokai University School of Medicine because the cystic component of the tumor was increasing in size. His past medical history was unremarkable, although his elder sister had been treated for leukemia.

No abnormality was detected upon physical examination. The results of laboratory tests, including those measuring tumor marker levels, were all within normal limits. The serum levels of insulin, gastrin, and glucagon were also within their respective reference ranges.

**Figure 1:**
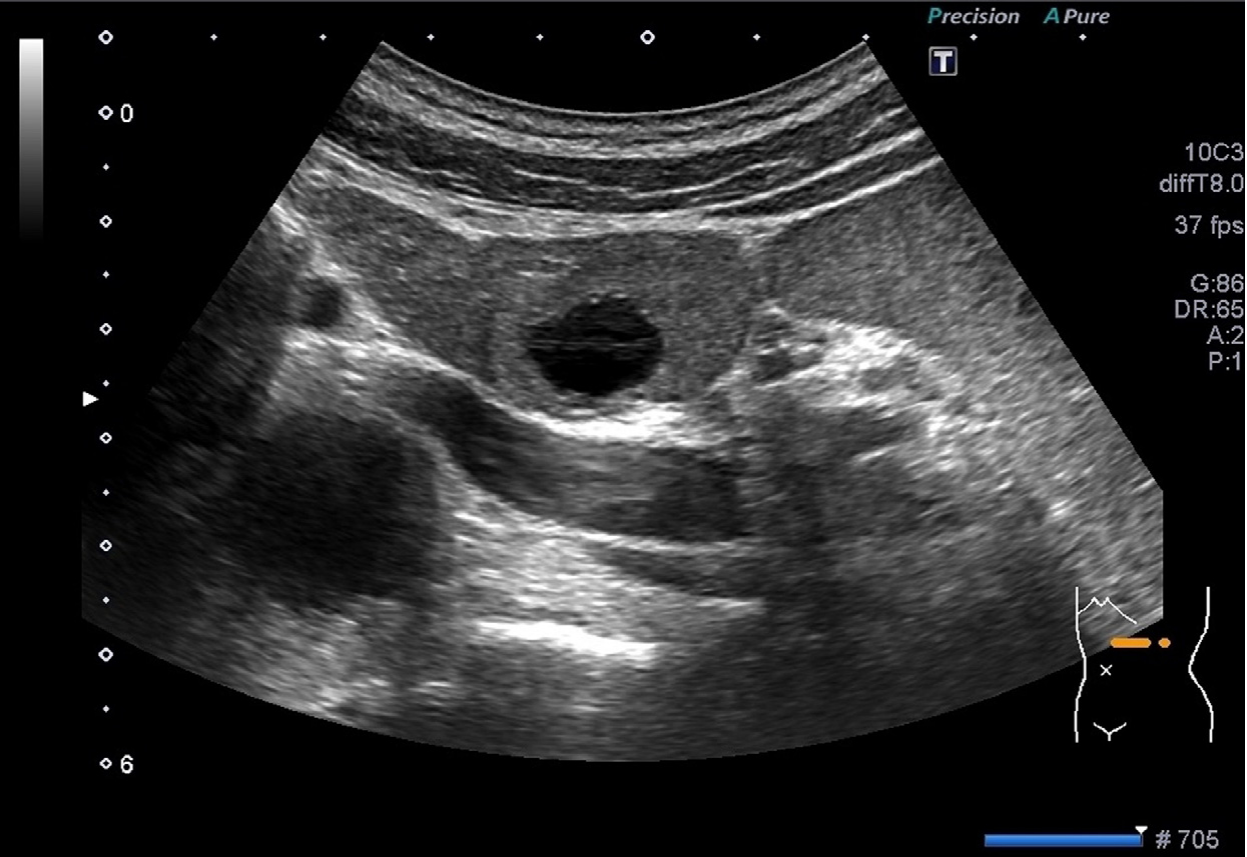
Abdominal ultrasonography revealed a 25‐mm cystic tumor located in the pancreatic tail.

**Figure 2:**
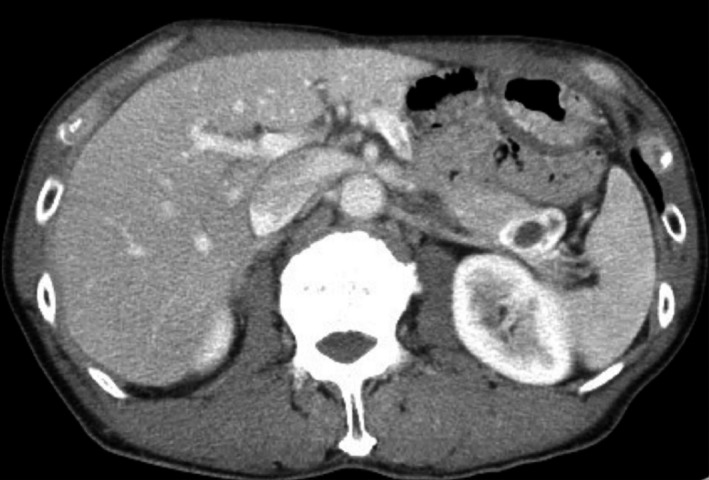
A cystic pancreatic endocrine neoplasm in the tail of the pancreas with an enhanced periphery.

**Figure 3:**
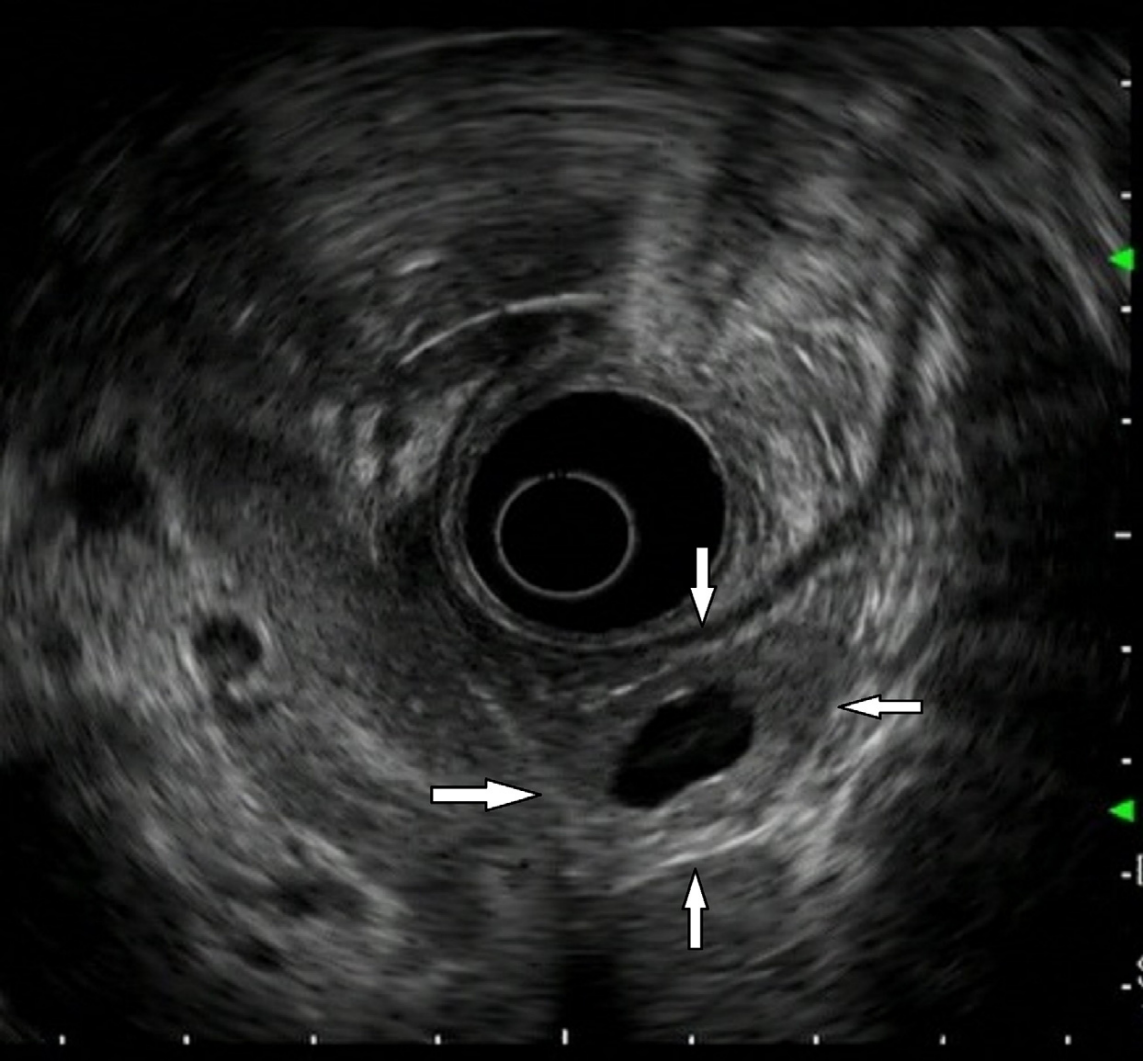
Endoscopic ultrasonography revealed a 25‐mm cystic lesion (white arrows) in the pancreatic tail.

Abdominal ultrasonography showed a well‐circumscribed tumor approximately 25 mm in size in the pancreatic tail, with a central cystic component approximately 10 mm in size (Fig. 1). The parenchymal portion of the tumor was uniformly rendered. In the portal phase, the cystic component was in the center of the tumor, which was well circumscribed with hyperstained margins (Fig. 2). Endoscopic ultrasonography also showed a 25‐mm cystic lesion with well‐circumscribed borders in the pancreatic tail region (Fig. 3), and magnetic resonance imaging confirmed this finding. Based on the above findings, a diagnosis of CPEN of the pancreatic tail was made.

Laparoscopic splenopancreatectomy was performed. A camera port was inserted into the lower abdominal region using the open technique, with the patient in the right half‐side‐lying position and the legs apart. Insufflation was started at 8 mmHg. Five trocars were inserted 5 mm above the midclavicular line, below the right and left costal arches (right: for the surgeon's left hand; left: for the assistant's right hand), 2 cm laterally and 5 mm to the right from above the midclavicular line at the naval level (for the surgeon's right hand), and 12 mm to the left of this point (for the assistant's left hand). After opening the omental bursa and dissecting the splenocolic ligament, a neoplasm was found in the pancreatic tail region near the splenic hilum. The base of the splenic artery was exposed and resected using a clip. At the same site, the pancreatic parenchyma was transected after slowly crushing it for 10 min using a powered ECHELON FLEX™ gold cartridge (Ethicon, Somerville, NJ). The pancreatic tail and spleen were isolated from the retroperitoneum and stored in E‐Z PASS (Hakko Co., Ltd., Chikuma‐shi, Nagano, Japan). The camera port incision site in the lower abdominal area was lengthened to 4 cm before extraction of the port. A drain was inserted into the pancreatic stump and surgery was completed. The operative time was 2 h 20 min. Only a small amount of blood was lost.

**Figure 4:**
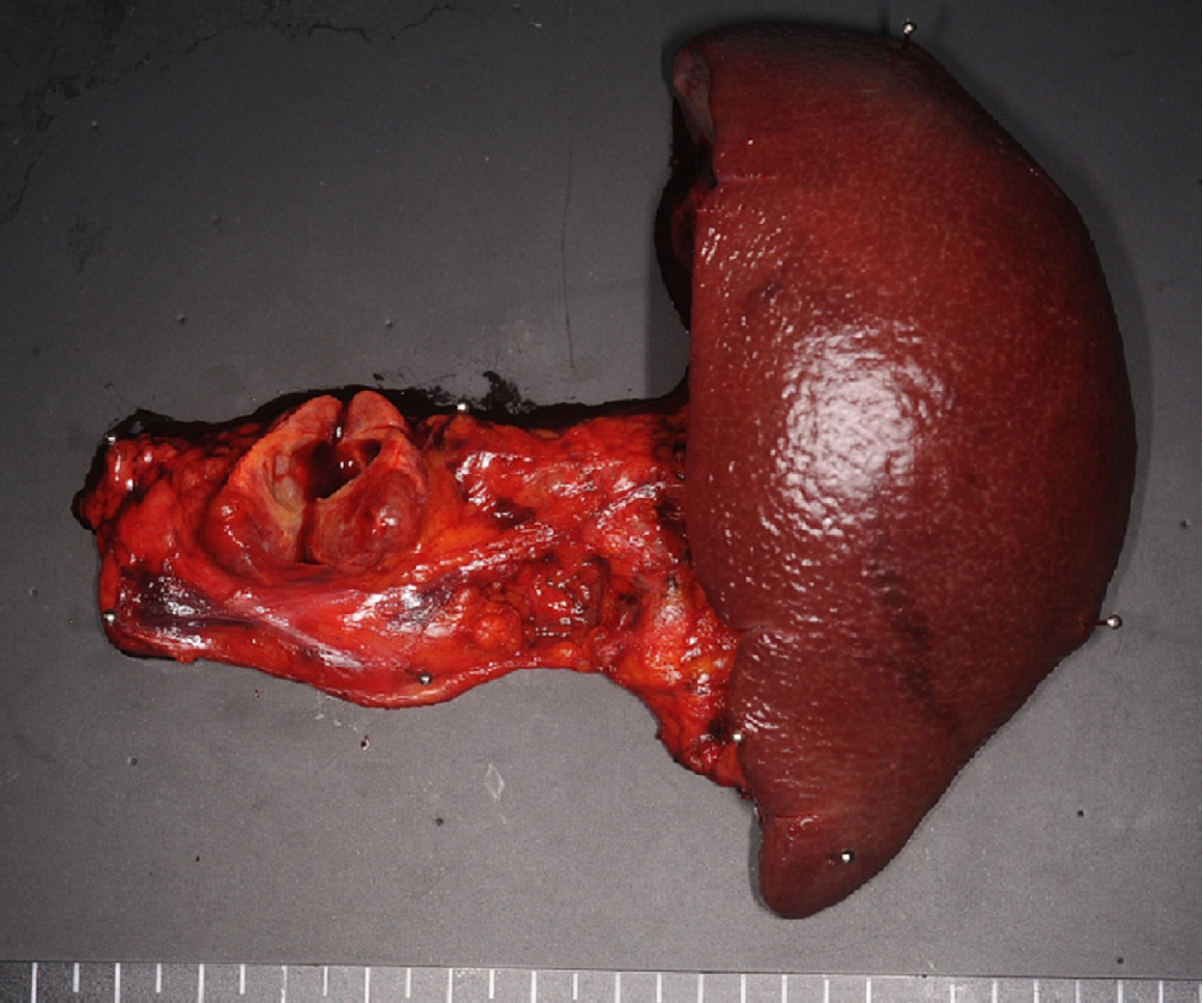
Macroscopic examination of the pancreatic cystic lesion.

**Figure 5:**
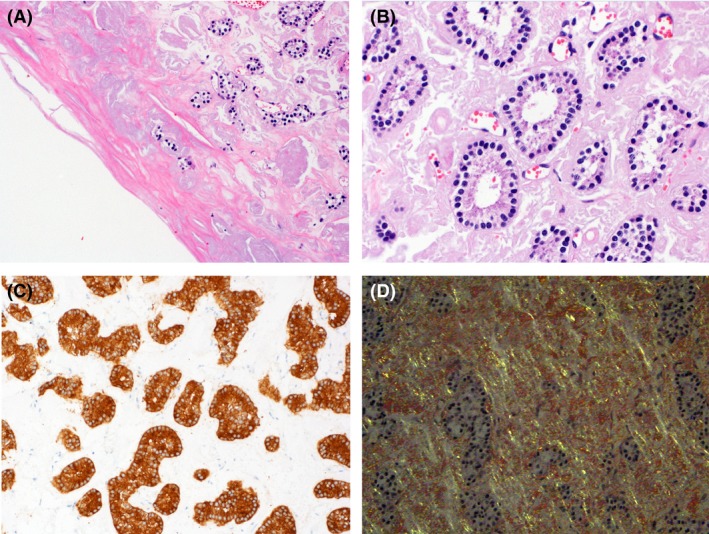
(A) There were no epithelial cells lining the cystic lesion. (B) Hematoxylin and eosin staining (magnification, 10 × and 20 × ). Immunohistochemical reactivity of the pancreatic endocrine neoplasm, which is positive for synaptophysin (C) and Congo red (D).

Macroscopically, a well‐demarcated, brownish, solid tumor with a unilocular cystic lesion was observed in the pancreatic body. The size of the tumor was 25 × 24 × 13 mm (Fig. 4). Microscopically, the tumor was composed of tumor cells within glandular or rosette‐like structures (Fig. 5A and B). The tumor cells had mildly enlarged nuclei with a “salt‐and‐pepper” chromatin pattern and eosinophilic, granular cytoplasm. Mitosis was not observed in 10 high‐power fields. A large number of eosinophilic and amorphous deposits were observed around tumor cell foci. When stained with Congo red and examined under polarized light, the deposits had an apple‐green birefringence, indicating they were composed of amyloids (Fig. 5D). The tumor cells and amyloid deposits were exposed to the cystic space. There were no epithelial cells lining the cystic lesion (Fig. 5A).

Immunohistochemically, the tumor cells were diffusely positive for synaptophysin (Fig. 5C), CD56, and somatostatin receptor 2A, weakly positive for chromogranin A, and negative for glucagon, pancreatic polypeptide, gastrin, somatostatin, and serotonin. Insulin was overexpressed in the tumor cells, but the patient experienced no symptoms associated with increased insulin levels. The MIB1 lab**e**ling index was 0.3%. From these findings, we diagnosed the lesion as a G1 neuroendocrine tumor. The tumor was compressing the main pancreatic duct; however, there was no observed invasion. There were no apparent lymph node metastases.

The postoperative course was uneventful, and the patient was discharged 7 days after surgery. The patient has been disease‐free for 14 months after undergoing the operation.

## Discussion

We discovered two important clinical issues: first, PENs usually appear radiologically as solid tumors but may rarely manifest as cystic lesions of the pancreas 2, and second, CPENs have a low potential for biological malignancy 6.

Pancreatic neuroendocrine neoplasms are a rare group of neoplasms with an estimated annual incidence of 0.4 per 100,000 7. However, their incidence is increasing, probably because of advancements in and increased use of radiographic and endoscopic imaging 8. PENs usually appear radiologically as solid tumors, although on rare occasions they manifest as cystic lesions of the pancreas. CPENs reportedly represent 7–17% of resected PENs 3, 4, 5, 9.

Opinions vary as to whether PENs should be treated via limited surgery, such as enucleation or middle pancreatectomy, or lymph node dissection. The diameter of a PEN is correlated to its degree of malignancy; in many reports, lymph node metastasis was often observed when the tumor diameter exceeded 2 cm 10, 11. Therefore, for PENs measuring ≥2 cm, standard surgery with dissection should be performed. However, as reported by Singhi et al. 12, lymph node and distant metastases are less common in CPEN cases than in PEN cases, as are nerve plexus permeation and vascular invasion. Moreover, CPENs have a low potential for biological malignancy 6, 12, 13 and, according to many reports, a better prognosis than PENs 3. Accordingly, limited surgery such as distal pancreatectomy without splenectomy and enucleation should be considered for CPENs that are not accompanied preoperatively by lymph node or distant metastasis, as was seen in the present case. Because the splenic artery contacted the tumor in our case, it was very difficult to leave it. Therefore, we selected splenopancreatectomy.

Cystic pancreatic endocrine neoplasm image findings include early dying effects in the solid part of the tumor, an oval and relatively thick cyst wall, and partitions and a protruding solid part in the lumen. Pancreatic cystic tumors requiring differentiation (and their frequencies) include intraductal papillary mucinous neoplasms (38%), mucinous cystic neoplasms (23%), serous cystadenomas (16%), and solid pseudopapillary neoplasms (3.7%) 9. In our case, the tumor had a cyst in its center as observed via endoscopic ultrasonography; it was well circumscribed, and the solid part exhibited uniform echogenicity. Therefore, we were able to establish a straightforward diagnosis of CPEN. For cases that are difficult to diagnose, endoscopic ultrasonography‐fine needle aspiration is useful and has a very high diagnostic accuracy 14, 15.

Cystic pancreatic endocrine neoplasm pathophysiology remains controversial, and several hypotheses have been proposed to explain CPEN formation 16. It was previously believed that as the tumor increased in size, vascular insufficiency occurred in its center, resulting in necrosis and subsequent cystic changes. Buetow et al. 17 reported that cystic changes were more likely to occur in tumors with larger diameters. However, owing to recent advances in imaging technology, cystic changes can even be observed in PENs ≤1 cm. Given the low frequency of necrosis in CPENs, Bordeianou et al. 5 speculated that necrosis is not the cause of cystic changes, but rather a secondary phenomenon. Moreover, according to Adsay et al. 14, the cyst wall is a sequence of endocrine cells, and the center of the cyst contains serous fluid rather than necrotic matter. In our case, there was little necrotic matter in the cyst, and the center of the cyst was filled with a dark brownish serous fluid. Upon histopathological examination, the cyst was positive for Congo red and DFS staining, indicating the presence of amyloid deposits in the cyst wall. A possible mechanism for the cystic changes observed in our patient is bleeding leading to chronic inflammation, which results in amyloid deposition.

In conclusion, the biological behavior of the CPEN was less aggressive than that of its solid counterpart. Accordingly, for cases of CPEN that are not accompanied preoperatively by lymph node or distant metastasis such as the present case, limited surgery should be considered. In our case, because of the short postoperative course, there is a need for follow‐up in the future.

## Consent

Written informed consent was obtained from the patient for publication of this case report and all accompanying images. A copy of the written consent form is available for review from the Editor‐in‐Chief of this journal.

## Conflict of Interest

The authors declare that they have no competing interests.

## Authorship

NY, DF, YM, MY, TM, and TN: performed surgery and postoperative management. YK, MO, YK, and TM: performed medical diagnoses. KH: performed the pathological diagnosis. All authors read and approved the final manuscript.
